# Next Generation Characterisation of Cereal Genomes for Marker Discovery

**DOI:** 10.3390/biology2041357

**Published:** 2013-11-25

**Authors:** Paul Visendi, Jacqueline Batley, David Edwards

**Affiliations:** 1 Australian Centre for Plant Functional Genomics, School of Agriculture and Food Science, the University of Queensland, Brisbane, QLD 4072, Australia; E-Mail: paul.muhindira@uqconnect.edu.au; 2 Centre for Biotechnology and Bioinformatics, College of Biological and Physical Sciences, the University of Nairobi, P. O. Box 30197 G.P.O, Nairobi 00100, Kenya.; 3 Centre for Integrative Legume Research, School of Agriculture and Food Science, the University of Queensland, Brisbane, QLD 4072, Australia; E-Mail: j.batley@uq.edu.au

**Keywords:** sequencing, single nucleotide polymorphisms, genotyping by sequencing, polyploidy, markers, cereals

## Abstract

Cereal crops form the bulk of the world’s food sources, and thus their importance cannot be understated. Crop breeding programs increasingly rely on high-resolution molecular genetic markers to accelerate the breeding process. The development of these markers is hampered by the complexity of some of the major cereal crop genomes, as well as the time and cost required. In this review, we address current and future methods available for the characterisation of cereal genomes, with an emphasis on faster and more cost effective approaches for genome sequencing and the development of markers for trait association and marker assisted selection (MAS) in crop breeding programs.

## 1. Introduction

**Figure 1:**
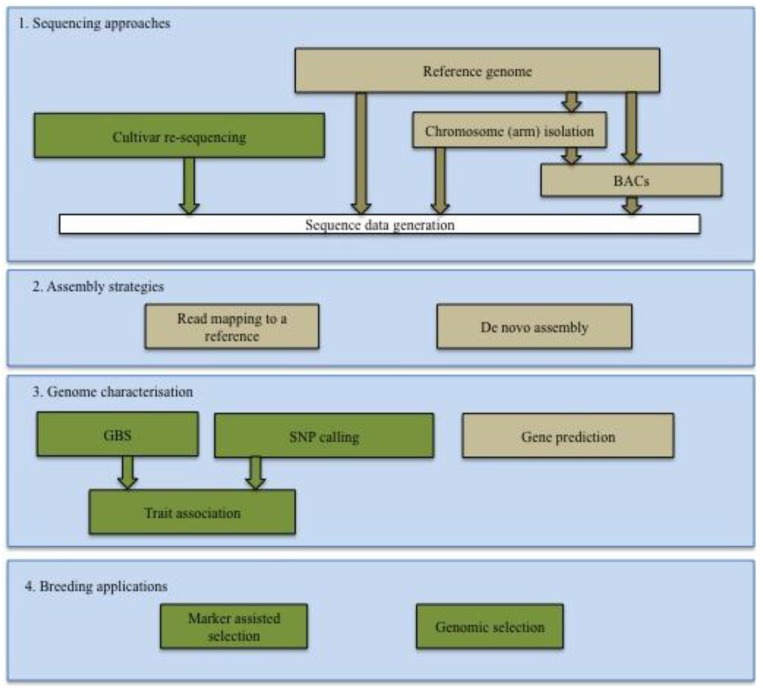
A schematic representation of cereal crop improvement using Next Generation Sequencing (NGS) technologies. Blue denotes main steps in the characterization of cereal genomes, brown denotes reference specific approaches while green represents applications to several cultivars or populations for variation discovery. (1) Sequencing approaches are determined by the project aims. For characterization of previously un-sequenced genomes without a closely related species, generation of a reference genome is undertaken. This may involve direct whole genome shotgun (WGS), chromosome (arm) isolation or BAC-by-BAC approaches or a combination of these. For GWAS, where a suitable reference genome is available, a large number of cultivars or populations are sequenced at low coverage. (2) Assembly strategies depend on the nature of the genome to be assembled, reads available (length, read types *i.e.*, paired end (PE) or mate pair (MP)), coverage depth, and whether there is a high quality draft genome of a closely related species of which if absent, de-novo assembly is undertaken. (3) Characterization then follows which involves gene prediction based on orthologous genes in related species or *ab-initio*. (4) Variation discovery through SNPs discovery and GBS within cultivars or populations enables trait associations and the generation of molecular markers for applications in crop breeding programs.

Cereals constitute over 60% of the world’s food sources. In the African continent, cereals comprise 46% of the diet, roots and tubers 20% and animal products 7%, while in Western Europe these constitute 26%, 20% and 4%, respectively (www.FAOstat.fao.org). The importance of cereals can be attributed to their phenotypic plasticity, enabling them to adapt to various climatic conditions. Several of the major cereal genomes are large and complex, mainly due to an abundance of transposable elements (TEs), and polyploidy [1,2]. As a result, genetic analysis of diversity, allele and haplotype frequencies is a challenge. Traditional breeding practices rely on phenotypic selection with cycles of 5–12 years depending on the crop and breeding system, however more rapid selection systems are urgently required to develop cereal varieties that are high yielding and resilient to floods, droughts and high or low temperatures to feed the growing world population in the face of climate change. The field of genomics is accelerating through the development and application of Next Generation Sequencing (NGS) technologies coupled with advanced computational algorithms and statistics. The cheaper per base cost of NGS compared to traditional Sanger sequencing comes at a cost of shorter read lengths and reduced accuracy, but offers the potential for increased depth of coverage required for confident variant discovery [3,4]. A summary of genomic approaches for crop improvement is presented in Figure 1. 

## 2. DNA Sequencing Technology

DNA sequencing technologies have evolved rapidly since the popular method developed by Sanger in the 1970s [5,6]. The initial Sanger sequencing method was automated [7] with improvements in read length and accuracy [8], resulting in error rates of as low as one in 10,000 bp, with read lengths between 800–1000 bp. Sanger sequencing is being rapidly replaced by NGS technologies. The first commercially available NGS platform was the GS20, produced by 454 Life Sciences and commercialised by Roche [9]. The latest 454 platform, the GS FLX+ model produces up to 700 Mbp per run, with read lengths of 1,000 bp. A major limitation of this pyrosequencing is the accurate determination of homopolymer regions. Illumina (www.illumina.com) have developed a range of popular NGS platforms and now dominate the NGS field. They apply a sequencing by synthesis (SBS) approach [10] and can produce read pairs where two reads are in a known orientation and approximate distance to each other, greatly facilitating genome assembly and read mapping in complex genomes. Their current platforms include the HiSeq systems which produce around 600 Gbp per run with read lengths of up to 150 bp; and the MiSeq which produces reads up to 250 bp within 24 hours, but with reduced data output of around 10 Gbp per run. The use of indexed paired read libraries, high data output and relatively low error rates makes this an increasingly popular technology for diversity studies, re-sequencing and SNP discovery [11,12,13,14,15,16]. 

Recent developments in third generation sequencing platforms (TGS) promise longer read lengths and eliminate bias caused by PCR amplification. Ion Torrent’s non-optical DNA sequencing technology (www.iontorrent.com) is based on complementary metal-oxide semiconductors (CMOS) [17]. Read lengths of 100–200 bp have been produced on a single run using 1.2 million sensors, generating more than 10 Gbp. The reduced cost and ease of scalability makes this technology cost-effective for re-sequencing and SNP discovery, though sequence error has yet to be fully evaluated. 

Pacific Biosciences (www.pacificbiosciences.com) apply a single-molecule sequencing technique called SMRT™ (Single Molecule Real Time) technology [18] in which nucleotides incorporated during synthesis are detected directly by DNA polymerase. Read lengths of 2,500–10,000 bp have been reported [19]. A drawback of these longer read lengths is increased error rates. Attempts have been made to correct these errors by using Illumina reads which are shorter but more accurate [20].

Oxford Nanopore (www.nanoporetech.com) exploits a synthetic protein with an ion channel at its core, embedded into a lipid bilayer membrane. Chauffer enzymes are utilised to either direct DNA strands into the protein nanopore (strand sequencing) or attach the DNA followed by cleaving one base at a time (exo-nuclease sequencing). In both cases, as nucleotides pass through the nanopore, specific disruptions to the current applied to the lipid bilayer are detected, enabling the determination of the DNA sequence of a strand [21,22]. While this technology is actively under development with little publicly available data on error profiles, Oxford Nanopore have reported error rates of about 4%.

### 2.1. Sequencing of Cereal Genomes

Rice was the first cereal to be sequenced [23], which paved the way for NGS characterization of more complex cereals. Bread wheat has a hexaploid genome (2n = 6x = 42) that contains three closely related ancestral diploid genomes (AABBDD), each with a set of seven chromosomes. The genome of bread wheat is also very large, around 17 Gbp and is predominantly composed of repeats [24,25]. Maize is an allotetraploid consisting of ~ 85% repeat sequence [26,27]. This compares to a repeat content of 35% in rice [23] and 55% in sorghum [28]. Due to the size and complex nature of most cereal genomes, sequencing, assembly and characterisation has been a daunting task. These challenges have led to the application of diverse approaches and sequencing platforms, such as BAC-by-BAC approaches, and the use of isolated chromosome arms [29]. 

Several attempts are currently underway to sequence the bread wheat genome. A recent whole genome shotgun (WGS) approach applied 454 sequencing technology, building an assembly of genic regions based on orthologous relationships to barley, sorghum, rice and Brachypodium [25,30]. With a WGS approach, the differentiation of homoeologous chromosome sequences is challenging. This complexity can be resolved by using flow cytometry to isolate individual chromosome arms [31] enabling a detailed study of homoeologous genes and translocations within wheat chromosome arms [32,33,34,35,36]. A BAC-by-BAC approach has also been applied to sequence isolated wheat chromosomes, with recent success for chromosome 3B. 

Both WGS and BAC-by-BAC approaches have also been combined to sequence other cereal genomes (Table 1). Rice, *Oryza sativa* ssp. *japonica* cv. Nipponbare, was sequenced by the International Rice Genome Sequencing Project (IRGSP) using a BAC-by-BAC approach based on genetic maps, BAC and YAC physical maps [23]. The resultant assembly included two earlier draft genome assemblies of rice from Monsanto [37] and Syngenta [38] that were sequenced using a WGS approach. The US Department of Energy (DOE) and the Joint Genome Institute (JGI) have sequenced the *Sorghum bicolor* genome using a WGS approach and validated the resultant assembly with 27 individually sequenced BACs [28]. The integration of physical and genetic maps with a BAC-by-BAC approach has also been used to sequence maize using a minimum tilling path (MTP) of 16,848 BACs and 63 fosmids [27]. A similar physical map has also been generated for barley [39]. 

Several factors impact the outcome of a genome assembly. These include; sequence coverage, data quality, repeats in the target genome and sequence read lengths. Sequence coverage and data quality are addressed by current sequencing platforms which produce large volumes of data cost effectively with high read accuracy, though there is a potential bias in base calling [40]. Different sequencing technologies have different error profiles, with 454 sequencing tending to exhibit homopolymer length errors, while Illumina base calling errors tend to occur towards the end of reads. Furthermore, different assembly methods result in different impacts of errors, with de Brujin graph methods handling sequence errors in Illumina short read data well, due to the relatively high k-mer coverage, compared to overlap layout consensus approaches frequently used for longer 454 and Sanger reads. 

**Table 1:** Current sequenced cereal genomes. All assemblies are usually shorter than the predicted genome size.

Crop	Assembly/Genome Size (Mb)	Year	Sequencing strategy	Reference
*Oryza sativa ssp. japonica* (Nipponbare)	370/389	2005	Sanger, BAC-by-BAC	[23]
*Oryza sativa ssp. japonica (Nipponbare)*	389/420	2002	Sanger, WGS	[38]
*Oryza sativa ssp. indica*	362/466	2002	Sanger, WGS	[191]
*Setaria italica (Foxtail Millet)*	423/515	2012	Illumina, WGS	[192]
*Sorghum bicolor (L.) Moench*	679/730	2009	Sanger, WGS	[28]
*Zea mays (Palomero Toluqueno) (popcorn)*	177/2100	2009	Sanger, WGS	[193]
*Zea mays (B73)*	2000/2300	2009	Sanger, BAC-by-BAC	[27]
*Triticum aestivum (Bread wheat)*	*/17000	2012	454, WGS	[25]
*Hordeum vulgare (Barley)*	4900/5100	2012	454, BAC-by-BAC	[194]
*Aegilops tauschii*	4491/4630	2013	Illumina, 454, WGS	[195]
*Triticum urartu*	3920/4940	2013	Illumina, WGS	[196]

* The *Triticum aestivum* assembly was that of orthologous genic sequences.

Repeats, either due to transposons, centromeric regions, ribosomal genes or polyploidy affect the quality of sequence assembly, and their impact is also dependent on the assembly algorithm applied. For many genomes, and especially highly repetitive cereal genomes, repeats pose the greatest challenge to attaining accurate assemblies. Long read lengths that span repeats would be desirable, but the current main NGS sequencing platforms have read length limits of 1 kbp. Greater read lengths can be obtained with some third generation sequencing technologies, but with these, sequence quality is compromised and they still would not span the extensive repetitive regions observed in many cereals. As such, a significant shortfall of current sequencing and assembly methods is the poor resolution of repeats, often resulting in collapsed repeats [40,41] within assemblies. The application of mate pair (MP) sequence data, where reads are several kbp apart, improves the resolution of repeats, and this has greatly expanded the scope of WGS genome assembly projects. It is expected that read lengths and MP technology improvements will continue to enhance the application of NGS technologies for sequencing complex cereal crop genomes. 

## 3. Genome Characterization

### 3.1. Orthology and Synteny Based Characterisation

Marker development is greatly dependent on access to well characterised reference genomes from which gene prediction, annotation and trait association follows. For cereal genomes without well-characterised reference genomes, gene orthology to closely related species can be used to assist in gene prediction and annotation. Gene orthology is a generally accepted approach to infer gene function for genes of newly sequenced genomes sharing an ancestor with a well-characterised reference. However, recent studies have showed that orthologous relationships do not necessarily imply functional equivalence, specifically in the context of complex evolutionary history, as reviewed in [42]. 

Cereal genomes exhibit complex evolutionary histories, and as such, orthology based synteny is currently the preferred approach to functional annotation of novel cereal genomes. Such approaches in wheat using isolated chromosomes and chromosome arms 3B, 4A, 4BS, 4D, 5A, 5D, 7BS, 7DS [32,33,35,43,44,45,46,47,48,49,50,51,52,53] are based on synteny conservation with multiple closely related grasses such as rice (*Oryza sativa*) [23], sorghum (Sorghum bicolor) [28] and Brachypodium [54]. Rice and Brachypodium have ~80% of their genes in conserved syntenic positions, Brachypodium being the closest relative to the *Triticeae,* having diverged around 25–30 million years ago (MYA), while ~40 MYA, divergence between rice and Brachypodium occurred, and sorghum diverged earlier at ~50 MYA [54,55,56]. As such, wheat and Brachypodium have more than 80% of their genes being syntenic [32]. Despite the success in the use of synteny for annotation of genes, the identification of non-syntenic genes remains a challenge. Exploiting multiple synteny observed among the *Triticeae* and leveraging on previous genomic studies still remains useful as it gives greater confidence in functional inference and trait association and continues to be applied to cereal genomes. 

### 3.2. Single Nucleotide Polymorphisms (SNPs)

Traditional marker systems such as restriction fragment length polymorphism (RFLPs) [57] have been applied in wheat [58,59,60,61], rice [62,63,64,65], barley [66,67,68,69], sorghum [70,71,72,73] and maize [74,75,76]. RFLPs were replaced by amplified fragment length polymorphisms (AFLPs) [77] and simple sequence repeats (SSRs) [78], which in turn have mostly been replaced by single nucleotide polymorphisms (SNPs) [79,80,81]. AFLPs have been widely applied to cereals including maize [82,83,84,85,86,87,88,89], sorghum [90,91,92], barley [93,94,95,96,97] and wheat [98,99,100,101,102], while SSRs have been exploited for diversity studies in sorghum [103,104], rice [105,106,107,108], wheat [104,107,108,109,110], maize [90,111], soybean [112] and millet [113]. SSRs have also been successfully used for genetic mapping studies in several cereals such as Tef (*Eragrotis tef*) [114], sorghum [115], soybean [116,117,118], maize [117], rice [119], wheat [120,121,122,123,124,125,126,127], rice and wheat [128] and millet [129,130]. Additionally, SSRs have been used for mapping of complex traits, for example in wheat [53,131,132,133]. SSRs have also been mined from ESTs [134,135,136,137,138], though EST based SSRs have been shown to have lower polymorphism when compared to genomic SSRs [104,109]. Despite this, EST SSRs have been applied across cereals [128,139]. 

SNPs are now the most common form of marker for genetic analysis [140,141,142]. They are abundant in plant genomes and their abundance provides very high resolution compared to other markers [104,109]. SNPs can be categorised as transitions or transversions [143,144]. Transitions are where the differing nucleotides are both purines (A/G) or both pyrimidines (C/T). When the SNP is between a purine and a pyrimidine, (C/G, A/T, C/A, or T/G) the SNPs are categorised as transversions. While indels are not true SNPs, they are sometimes considered as SNP markers, as they can be assayed in the same way as SNP markers. 

Given the prevalence of genome duplication in plants [145,146], and specifically cereals [147], SNP identification is often confounded due to homoeologous and paralogous genes. This genome complexity makes SNP discovery a significant challenge. For example, about 40% of SNPs predicted in maize have been attributed to paralogous genes [27,148]. In addition to genome complexity, the high rate of sequence error in NGS data generates a further challenge for SNP discovery. Several approaches have been used to assess and improve SNP calling accuracy, these include a SNP redundancy score, which is a count of how frequently a SNP is observed at a particular locus [149], and the transition/transversion ratio can also be used to provide an indication of the overall SNP prediction accuracy. This is as a result of higher mutation rates observed in methylated C nucleotides [150], although other mechanisms such as UV radiation are also thought to contribute [151]. 

The large data volumes produced by Illumina sequencing enables the identification of high-density SNP markers, potentially driving genomics assisted crop improvement in complex crops, such as wheat, in the future [152,153] and further revolutionising genotyping by sequencing (GBS) approaches. This is evident in wheat where more than 900,000 SNPs have been identified on the group 7 chromosomes with 93% validation accuracy [154,155], and 14,078 SNPs identified from 6,255 distinct wheat reference sequences with a 65% validation rate [156]. Similar approaches to SNP discovery using Illumina data have also been successful in rice, with the identification of 3.6 million SNPs from 517 rice landraces, providing a model for complex trait association [157], and more than 1 million SNPs identified between six inbred maize lines [158]. 

Several tools have been developed for the discovery of SNPs from plant NGS data [159,160,161,162,163]. These include AutoSNPdb, which determines SNPs from 454 transcriptome data [164,165,166] (http://www.autosnpdb.appliedbioinformatics.com.au/) storing results in a relational database for web based querying. AutoSNPdb is based on autoSNP software which scores SNPs based on redundancy score and co-segregation [149,167]. Second-Generation Sequencing autoSNP (SGSautoSNP) has been applied to identify more than 1.5 million SNPs in canola, with accuracy greater than 95% (D. Edwards, unpublished data) with similar success in wheat with an accuracy of greater than 93% of SNPs being between wheat cultivars [154]. Other approaches involve targeted genomic SNP identification [168], and AGSNP, which has been applied to identify 497,118 candidate SNPs in *Ae. tauschii* [169]. Some of the identified SNPs have been applied for the development of high throughput Illumina Infinium assays, for example in barley [170], wheat [171], canola and maize [172] .

### 3.3. Genotyping by Sequencing (GBS)

Genotyping by sequencing (GBS) extends traditional approaches to genotyping by exploiting NGS technologies to calling genotypes. The first published GBS approach [148] involved the use of 27 inbred maize lines, reducing the complexity of the genome with methylation sensitive restriction enzymes followed by sequencing and mapping the reads to the B73 maize reference genome [173]. Polymorphic sites among the inbred lines were then determined which showed evidence for specific regions involved in domestication and the geographic adaptation of maize. Similar approaches have recently been applied to 50 rice accessions [174]. This study identified candidate domestication genes that had low diversity in the cultivated rice accessions compared to wild type accessions. Two well-known rice domestication genes, *prog1* [175,176] and *sh4* [177], associated with erect growth and pod shattering, respectively, were identified. The main advantage with this approach over other genotyping methods is that no predetermined markers are required to study a particular population, as the markers are developed during the genotyping. Such approaches have been successfully demonstrated in rice, both with parental lines [178,179] and without the use of parental lines [180], as well as more recently in durum wheat [181].

The high marker density associated with GBS makes it a suitable platform for genome wide association studies (GWAS). A recent study in *Arabidopsis arenosa* [182] in which 12 *A. arenosa* individuals selected from Austria and Germany were sequenced, identified selective sweeps within the genome and indicated genes associated with housekeeping processes such as chromosome segregation, cohesion, transcription regulation and homologous recombination which were active as a result of genome duplication. In particular, a non-synonymous mutation in the meiosis gene *ASYNAPSIS1* was identified as a rare variant in diploid *A. arenosa,* highlighting ongoing mutations in the diploid genome. A larger study in rice [157] in which 517 rice landraces of *Oryza sativa indica* subspecies were sequenced with subsequent GWAS analysis of 14 agronomic traits, showed approximately 36% of the identified loci explained phenotypic differences.

The advent of NGS technologies and associated reduction in sequencing costs has made skim based genotyping by sequencing, without complexity reduction, feasible. Skim GBS offers advantages over other genotyping by sequencing methods in that it is genome wide with flexible density determined by the quantity of data generated. Other GBS approaches rely on targeting specific regions on the genome. Such approaches include the use of complexity reduction of polymorphic sequences (CRoPS) methods as shown in maize [183,184] and wheat [185], the use of restriction enzymes followed by sequencing in mapping populations in wheat, maize and barley [146,186,187]. 

As GBS approaches offer quicker and more accurate recombination breakpoint determination, with higher accuracy and resolution due to high density, more individuals can be analysed at a relatively lower cost. As DNA sequencing costs continue to decline, it is expected that GBS without the bias of complexity reduction will become increasingly popular for cereal genome analysis.

## 3. Conclusions

As more cereal genomes are sequenced, storage and analysis of this vast amount of data has been an increasing challenge, though this challenge has been met with advances in bioinformatics [188]. With further improvements to sequencing platforms resulting in longer reads, combined with the expansion of third generation single molecule sequencing technologies, genome sequencing GBS and GWAS are likely to increase in popularity. As an increasing number of cereal crop genomes are sequenced, there will be a move away from the generation of genome references and a greater focus on trait association, leading to a greater understanding of the function of these genomes on a population scale and bridging the genotype to phenotype divide [189] with insights into the emerging concept of the ‘Pangenome’ [190] in the context of crop breeding and improvement. 
